# Among the Colleges

**Published:** 1896-01

**Authors:** 


### AMONG THE COLLEGES.

ONTARIO VETERINARY COLLEGE. This institution held its Christmas examination on December 20th. The usual Board of Examiners, which is composed of practising veterinary surgeons, officiated. The following gentlemen, after being subjected to a rigid examination, were awarded graduation diplomas: Donald Currie, Stayner; Francis Duncan, Unionville; John M. Farquhar, Greenock, Scotland; Truman Earl Gore, Clarksburgh, W. Va., U. S.; Cecil Howell, London, Ont.; John S. Jones, Poland, N. Y.; R. L. Kann, Lisburn, Pa.; Archie A. McArthur, Stayner; Allan McDonald, Erin; Angus McDonald, Teeswater; John J. McGregor, Carleton Place; Arthur E. Miller, Myersville, Ohio; James H. Powers, Providence, R. I.; David D. Reid, Teeswater; William J. Rouse, Mitchell Square; Daniel Henry Super, Warrensville, Pa.; Jacob W. Wagner, Tavistock; E. C. Wisman, Bryan, Ohio.

Primary examination, anatomy—George Hamilton Leslie.

UNITED STATES COLLEGE OF VETERINARY SURGEONS. This school has materially strengthened its faculty and added to the advantages offered her students by securing for the chair of zoötechnics and sanitary medicine the services of Dr. J. P. Turner, graduate of the Veterinary Department of the University of Pennsylvania and veterinarian to the Sixth Regiment of the United States Army.

The chair of microscopy and bacteriology will in the future be filled by Walter W. Alleger, Ph.D., M.D.

A VETERINARY college, carrying the royal seal, is now assured for Ireland, and will be located at Dublin. A grant of some seventy-five thousand dollars will be accorded by Parliament for the establishment of suitable buildings.

THE NEW YORK COLLEGE OF VETERINARY SURGEONS has a junior class of twenty-three under the new order of a three years' course. In her three classes some fifty-four students are in regular attendance.

MCKILLIP VETERINARY COLLEGE has some twenty-three students enrolled in her two classes, a very good showing for a new school entering upon its career at a time when every other college is suffering a serious loss in the number of her matriculants. Starting out as a three years' school, and having raised her tuition fee from seventy-five to one hundred dollars the second year, would strongly indicate that the future veterinarian appreciates the fact that he must be better educated than his predecessor to win a livelihood and place among scientific workers.

OHIO VETERINARY COLLEGE. Those directors and stockholders of the Ohio Veterinary College at Cincinnati who were induced to invest their money in the establishment of this school have found it rather a serious undertaking, and not so great a money-winning venture as they supposed. They are very willing to sell out at the present time at a great shading of the original value of the stock, and the directors and stockholders are now confronted with lawsuits for the debts contracted in the name of the school, and are endeavoring to evade payment of the same. They no longer direct the affairs of the school, but have leased the building, good-will, etc., to Dr. Thomas King, the former dean, whom we understand assumes all responsibilities from October 1, 1895, and who has in view the purchase of the franchise and the reorganization of the school next year. There can be but one outcome for a long period in the future of such schools, erected on the basis of a money-winning investment—they will go to the wall, for the time to-day required in properly equipping a veterinary practitioner is very different from ten years ago, and this means a large outlay of money for equipment and maintenance that cannot be won back for many years. This school in its short but varied career has changed her dean three times, and each contributed largely to a forced rearrangement of her classes and studies.

All students matriculating after January 1, 1896, must do so for a three years' course, which adds another school to the advanced colleges.

CHICAGO VETERINARY COLLEGE has suffered a serious loss in the number of her students for the present year. Her total number of matriculants falls below fifty. The hard times throughout the whole country, and especially the West, have told severely upon the classes in all veterinary colleges.

THE REFORM OF VETERINARY STUDIES IN AUSTRIA. On the 26th and 27th of September discussions were held by the Ministry of Education in regard to the reform of veterinary studies. There were present at the meeting representatives from the Department of the Interior (Dr. Emanuel R. von Kusy and Bernard Sperk), from the Department of Education, from the Department of Agriculture (Freiken von Hohenbruck), from the Department of War (Colonel von Huber), from the Medical Faculty in Vienna (Prof. Max Gruber), from the Faculty of the Imperial Military Veterinary Institute (Dr. Bayer and Professor Polansky), from the Faculty of the Lemberg School (Professor Kadyi and Dr. Szpelman), from the Faculty of the Agricultural High School (Professor Nieckeus), and finally from the Veterinary Association of Austria (Dr. Hausner from Feldsberg).

According to the meagre reports of the preceedings and result of the discussions which have come to us the Commission gave serious consideration to some of the suggestions which were made a long time ago by the Veterinary Association of Austria, so that now there is hope that the Austrian veterinary schools will at no very distant day be put on a par with those of the neighboring States. We refer here especially to the requirement of greater maturity (on the part of the students), and what stands in intimate connection with the same, namely, the raising of both institutions to high schools. The lengthening of the course to four years and a thoroughgoing reform of the course of study, with especial regard to practice, would follow as a matter of course, and not only the oft-expressed wishes of veterinarians, but also those of agriculturists, would be realized, in so far as the latter do not belong to those circles who consider it opportune to plead for the lowering of the standard of education among veterinary surgeons.

The discussion was held over a very comprehensive and elaborate plan of study, the details of which received the most thorough and objective criticism, at the same time the difference in the standpoints of the civil and military departments were brought out clearly, although these differences were evidently not of enough importance to lay any obstacles in the way of the reform aimed at. We cherish the hope, therefore, that the era of reform already begun may not be hindered in its progress by these differences of opinion, and that the question of finances may not prove an obstacle.

The proposed plan of study, which reached us just before going to press, is as follows:

*First Year.* First Term: Physics and chemistry, as far as they relate to medicine; general zoology and parasites; general anatomy and histology; descriptive anatomy of the domestic animals; theory of horseshoeing and exercises in the blacksmith shop; exercises in preparing anatomical specimens. Second Term: Chemistry; botany, with especial reference to food, medical, and poison plants; encyclopædia of agricultural plants, more especially of the diseases of food-plants; descriptive anatomy of the domestic animals; embryology; exercises in the chemical laboratory; exercises in the use of the microscope and in the examination of normal tissues; exercises in the blacksmith shop; instruction and exercises in the handling of domestic animals, harnessing and saddling, and as far as possible in riding and driving.

*Second Year.* Third Term: Topographical anatomy of the domestic animals; theory of breeding (judging, training, feeding, etc.), of the agricultural animals; physiology; pharmacognosia; pharmacology; toxicology; theory and art of prescribing; preparation of anatomical specimens; exercises in the examination of food (food-stuffs); pharmaceutical exercises. Fourth Term: Theory of cattle-breeding; physiology; general and experimental pathology and pathological anatomy; bacteriology; pharma cognosia; pharmacology; toxicology and theory and art of prescribing; exercises in external examination and in judging the agricultural animals; excursions for the purpose of practical instruction in cattle-breeding; pharmaceutical exercises.

*Third Year.* Fifth Term: Special pathological anatomy of the domestic animals; special pathology and therapeutics and internal clinic; special surgery, together with ophthalmology and surgical clinic; theory of instruments, bandages, and operations; pathological operations; exercises in operating and in application of bandages; exercises in the use of the ophthalmoscope. Sixth Term: Special pathology and therapeutics and internal clinic; obstetrics; history of veterinary surgery; pathological operations; pathological, histological, and bacteriological exercises; practice in operating and in applying bandages; obstetrical exercises with the phantom and on living animals.

*Fourth Year.* Seventh Term: Special pathology and therapeutics and internal clinic; special surgery, together with ophthalmology and surgical clinic; theory of epidemics and demonstrations; State veterinary medicine (cattle- and meat-inspection, veterinary police, etc.); seminary for State veterinary medicine; pathological operations and specimens; polyclinic. Eighth Term: Special pathology and therapeutics and internal clinic; special surgery, together with ophthalmology and surgical clinic; polyclinic; ambulatory clinic in the city and neighborhood; temporary disposition of students in districts for the purpose of introducing them into practice; exercises and excursions for the purpose of instruction in State veterinary (sanitary) medicine; pathological operations.

*Examinations in Course.* Students of veterinary medicine can only be inscribed in the second- or third-year course after they have passed the examinations of the preceding year satisfactorily. The examination in course for promotion into the second year includes: Medicinal physics; chemistry; general zoölogy and parasites; botany; encyclopædia of the plant kingdom, with special regard to the disease of food-plants. The examination for entrance into the second year includes: Theory of animal breeding; anatomy of the domestic animals, including topographicpl anatomy; histology and embryology; physiology, pharmacology, together with pharmacognosia, toxicology, and prescribing.

*Final and More Thorough Examinations.* 1. Theory of animal breeding; descriptive and topographical anatomy of domestic animals; histology and embryology; physiology, pharmacology, with pharmacognosia, toxicology, and theory and art of prescribing. 2. General pathology and pathological zoötomy; bacteriology; special pathology and therapeutics and theory of epidemics; State veterinary medicine. 3. Surgery, inclusive of ophthalmology and theory of operating; obstetrics; horseshoeing. In the finals, the examination in every subject takes place at one time (in one act), and is practical as well as theoretical.

For the 1st final the candidate must pay a fee of 40 florins; for the 2d and 3d one of 35 florins each. In case of failure the candidate has only to pay half the sum at a second examination.

The veterinary diploma is written in the Latin language. For the preparation of the diploma upon parchment the fee is 10 florins, and for every subject in the examinations in course 3 florins.—*Thierärztliches Centralblatt*, October 1, 1895.

